# *Brain Sciences* Special Issue “Neuromodulation of Cortical Networks in Neurological and Neuropsychiatric Disorders: Potential Clinical Indications and the Biophysiological Impact of Stimulation”

**DOI:** 10.3390/brainsci14050459

**Published:** 2024-05-01

**Authors:** Maja Rogić Vidaković, Joško Šoda, Joshua Elan Kuluva

**Affiliations:** 1 Laboratory for Human and Experimental Neurophysiology, Department of Neuroscience, School of Medicine, University of Split, 21000 Split, Croatia; 2 Signal Processing, Analysis and Advanced Diagnostics Research and Education Laboratory (SPAADREL), Faculty of Maritime Studies, University of Split, 21000 Split, Croatia; jsoda@pfst.hr; 3 Piedmont Neuroscience Center, Oakland, CA 94610, USA; joshuakuluvamd@piedmontneurosciencecenter.com

Individuals with neurological and neuropsychiatric disorders face a variety of difficulties that can significantly impact their daily lives. Neuromodulation of cortical networks following electrical and magnetic stimulation techniques has been utilized as a therapeutic tool to assist in treating some of these conditions. Stimulating cortical networks can enhance neuroplasticity, act as a neuro-rehabilitative tool, or recalibrate aberrant neural pathways. The question is how brain stimulation results in lasting changes in cortical excitability, how brain dynamics evolve during stimulation, how we can quantify the excitability changes in human cortical networks, and how these changes translate into improved clinical outcomes. However, based on our knowledge of nervous system function and the potential benefits of neuromodulation, much more remains to be learned about how this type of intervention can be used to treat these disorders.

This Special Issue consists of a total of ten papers (six articles, two systematic reviews, one review, and one case report), covering topics on the use of the following: (a) high-frequency repetitive transcranial magnetic stimulation (rTMS) in investigating excitability of the corticospinal tract by combining motor training, rTMS, and trans-spinal magnetic stimulation (rSMS) in healthy subjects (Contribution 1); (b) rTMS (high- and low-frequency rTMS), theta burst stimulation (TBS), and transcranial direct current stimulation (tDCS) as non-invasive brain stimulation techniques (NIBS) in alleviating post-traumatic stress disorder (PTSD) symptomatology (Contribution 2); (c) rTMS (high and low-frequency) and tDCS (anodal tDCS, chatodal tDCS) over the dosal lateral prefrontal cortex (DLPFC) as NIBS techniques in investigating gambling disorder (GD) (Contribution 3); (d) high-frequency (10 Hz) rTMS over the DLPFC to study negative feelings of social anxiety (Contribution 4); (e) low-frequency (1 Hz) rTMS over the prefrontal cortex (PFC) and DLPFC to study mood change in healthy subjects with the personality influence (Contribution 5); (f) tDCS efficiency in insomnia treatment in animal model (mice) (Contribution 6); (g) tDCS investigating dizziness in healthy single male subject with vestibular migraine by provoking dizziness using a virtual reality device for four weeks and measuring functional near-infrared spectroscopy (fNIRS) quantitative electroencephalography (EEG), dizziness and visual vertigo standardized scales (Contribution 7); (h) single-pulse TMS to primary motor cortex for laryngeal muscle representation (M1) in investigating cortico-inhibitory processes (measure of cortical silent period, CSP) by recording motor evoked potentials (MEPs) and CSP from laryngeal muscles in laryngeal dystonia disease (prior called spasmodic dysphonia), a rare idiopathic disease of unknown cause (Contribution 8); (i) vagus nerve stimulation (VNS) in seven pediatric patients for treatment of refractory status epilepticus (RSE)/super refractory status epilepticus (SRSE) (Contribution 9); and (j) cognitive and seizure outcomes in drug resistant epilepsy patients with responsive neurostimulation (neurostimulator placed in the skull, under the scalp) and those who underwent left anterior temporal lobectomy (Contribution 10).

The preliminary studies provide the following evidence:

(a) rSMS increases excitability and motor training in healthy subjects, which gives future directions to investigate the efficacy of rSMS in patients who cannot be treated effectively with rTMS (Contribution 1).
(b) NIMBS reduces the severity of PTSD symptoms (Contribution 2). Future studies should investigate a combination of NIMBS with psychotherapeutic therapy.
(c) Furthermore, a NIMBS is a feasible treatment option for reducing GD symptomatology; however, caution is needed to yield conclusive results, and therefore, further studies are needed to validate the findings (Contribution 3).
(d) Moreover, a 10 HZ rTMS applied over the right hemisphere induces more social anxiety symtomatology (feelings of exclusion) (Contribution 4), which might suggest the possibility of increased feelings of social pain. Further studies are needed to control the sample size, the experimental task and design (i.e., game duration, questions), and investigate other brain areas (i.e., ventrolateral prefrontal cortex, insula, etc.).
(e) Utilizing a 1 Hz rTMS over the PFC was negatively correlated with sensation-seeking personality (Contribution 5), suggesting that individuals with higher levels of sensation-seeking may have a different response to rTMS compared to those with lower levels of sensation-seeking, which might be explained by a possibly more sensitive dopamine system in higher levels of sensation seeking subjects. However, the study results need to be interpreted with caution due to the following reasons: non-properly controlled use of the sham condition; use of a single 1 Hz rTMS protocol; construct used in the evaluation of subjective mood; and crossover design (1 Hz rTMS over the left DLPFC, PFC, and auditory cortex as the sham condition) executed consecutively in a single session per day, yielding a potential carry-over effect. The study findings contribute to the discussion on individual variability in personality and response to rTMS mood change.
(f) The effects of utilizing a tDCS (0.06 mA of electrical currents for 8 min) over the frontal lobe in mice on altering the quantity and duration of NREM sleep provide evidence for the involvement of the infralimbic area in insomnia (Contribution 6). However, the effects on REM sleep require further studies with a controlled sample size and additional experiments. In addition, stimulation positioning over the brain should be controlled in future tDCS studies investigating insomnia in mouse models.
(g) Furthermore, the application of a tDCS to provoke dizziness results in activation of the temporal cortices and excessive activation of CS, P3, and T5 in the left hemisphere and C4 in the right hemisphere (Contribution 7), and it could be considered a potential approach to investigating tDCS efficacy in alleviating dizziness in patients with vestibular migraine. However, further studies are needed.
(h) Utilizing single-pulse TMS studies on the neurophysiological mechanisms in laryngeal dystonia could yield specific rTMS protocols over the M1 for laryngeal muscle representation in treating laryngeal dystonia voice symptomatology (Contribution 8).
(i) On the early application of VNS in pediatric patients with RSE/SRSE (Contribution 9). The experimental results of the proposed study indicate that in five patients out of seven, the resolution of SRSE was observed after VNS implantation in the acute phase. Further studies are suggested on larger case series to prove the efficacy of early VNS implantation in RSE/SRSE patients.
(j) Drug-resistant epilepsy patients with responsive neurostimulation had higher seizure rates pre-intervention and a seizure frequency decline from pre- to post-intervention that were similar to those who underwent resective surgery (Contribution 10). Furthermore, patients with responsive neurostimulation and patients with right temporal lobectomy had similar neuropsychological outcomes. In contrast, left temporal lobectomy patients had deterioration in specific neuropsychological measures such as object naming and verbal learning. Further studies are needed on a larger sample of drug-resistant epilepsy patients to investigate their neuropsychological status, whether they are undergoing surgical resection or neuromodulation.

Taken together, the scope of the subject matter in the proposed Special Issue will, in all likelihood, be of interest to both new and experienced investigators involved in neurological, psychiatric, and psychological research, either preclinical or clinical. On this note, we thank all the authors who contributed to this *Brain Sciences* Special Issue, entitled “Neuromodulation of Cortical Networks in Neurological and Neuropsychiatric Disorders: Potential Clinical Indications and the Biophysiological Impact of Stimulation,” and lay the groundwork for further understanding of the neuromodulatory brain activity in various medical conditions disturbing brain pathways and networks.

